# Diagnostic accuracy of spleen stiffness to evaluate portal hypertension and esophageal varices in chronic liver disease: a systematic review and meta-analysis

**DOI:** 10.1007/s00330-020-07223-8

**Published:** 2020-09-24

**Authors:** Xing Hu, Xiaojie Huang, Jianhua Hou, Lei Ding, Chunling Su, Fankun Meng

**Affiliations:** 1 Ultrasound and Functional Diagnosis Center, Beijing Youan Hospital, Capital Medical University, No. 8, Xitoutiao, Youanmenwai, Fengtai District, Beijing, 100069 China; 2 Center for Infectious Disease, Beijing Youan Hospital, Capital Medical University, Beijing, China

**Keywords:** Elasticity imaging techniques, Spleen, Portal hypertension, Esophageal varices, Diagnosis

## Abstract

**Objectives:**

To systematically review studies on the diagnostic accuracy of spleen stiffness measurement (SSM) for the detection of clinical significant portal hypertension (CSPH), severe portal hypertension (SPH), esophageal varices (EV), and high-risk esophageal varices (HREV) in patients with chronic liver diseases (CLD).

**Methods:**

Through a systematic search, we identified 32 studies reporting the accuracy of SSM for the diagnosis of portal hypertension (PH) and/or EV in adults with CLD. A bivariate random-effects model was performed to estimate pooled sensitivity, specificity, likelihood ratio, positive predictive value (PPV), negative predictive value (NPV), and diagnostic odds ratios (DOR). The clinical utility of SSM was evaluated by Fagan plot.

**Results:**

A total of 32 studies assessing 3952 patients were included in this meta-analysis. The pooled sensitivities of SSM were 0.85 (95% confidence interval (CI), 0.69–0.93) for CSPH; 0.84 (95% CI, 0.75–0.90) for SPH; 0.90 (95% CI, 0.83–0.94) for any EV; and 0.87 (95% CI, 0.77–0.93) for HREV. The pooled specificities of SSM were 0.86 (95% CI, 0.74–0.93) for CSPH; 0.84 (95% CI, 0.72–0.91) for SPH; 0.73 (95% CI, 0.66–0.79) for EV; and 0.66 (95% CI, 0.53–0.77) for HREV. Summary PPV and NPV of SSM for detecting HREV were 0.54 (95% CI, 0.47–0.62) and 0.88 (95% CI, 0.81–0.95), respectively.

**Conclusions:**

Our meta-analysis suggests that SSM could be used as a helpful surveillance tool in management of CLD patients and was quite useful for ruling out the presence of HREV thereby avoiding unnecessary endoscopy.

**Key Points:**

• *SSM could be used to rule out the presence of HREV in patients with CLD thereby avoiding unnecessary endoscopy.*

• *SSM has significant diagnostic value for CSPH and SPH with high sensitivity and specificity in patients with CLD*.

• *SSM could be used as a helpful surveillance tool for clinicians managing CLD patients*.

**Electronic supplementary material:**

The online version of this article (10.1007/s00330-020-07223-8) contains supplementary material, which is available to authorized users.

## Introduction

Portal hypertension (PH) is a set of clinical syndromes caused by increased pressure in the portal venous system and is one of the primary consequences of chronic liver diseases (CLD), which can lead to the formation of extensive collateral circulation [1]. Clinical significant portal hypertension (CSPH) is defined as hepatic venous pressure gradient (HVPG) ≥ 10 mmHg, which could result in clinical complications of PH such as esophageal varices (EV), ascites, hepatic encephalopathy, and hepatorenal syndrome. Furthermore, severe portal hypertension (SPH) defined as HVPG ≥ 12 mmHg is a risk factor of variceal bleeding [2]. EV is the most important collateral circulation of PH and occurs in approximately 50% of cirrhotic patients, while variceal bleeding is associated with high mortality [3, 4]. Therefore, timely detection and accurate assessment are important in patients with PH and EV to ensure appropriate patient management.

HVPG and esophagogastroduodenoscopy (EGD) are currently considered the gold standards for evaluating PH and EV, respectively [5, 6]. However, measurement of the HVPG and EGD are invasive and potentially associated with complications, the application of the two types of detection methods is limited due to poor patient compliance [7]. In addition, the equipment used for HVPG measurement is demanding and requires professional technicians, so it is difficult to carry out routinely in clinical practice. Hence, alternative noninvasive techniques, with favorable diagnostic performance for evaluating PH and EV would be extremely attractive.

Elasticity imaging techniques including ultrasound elastography (USE) and magnetic resonance elastography (MRE) have been used to assess changes in spleen stiffness in various diseases [8]. Recent studies have shown that spleen stiffness is related to the progression of hepatic fibrosis, and in patients with hepatitis B/C infection, spleen stiffness is increased even though the liver stiffness is unchanged [9, 10]. Subsequent studies have demonstrated that spleen stiffness was positively correlated with HVPG and has good performance in predicting CSPH and EV in CLD patients [11, 12]. Other studies have indicated that although spleen stiffness is associated with PH, it is not sufficient to accurately assess the severity of PH [13]. Further studies have suggested that SSM could reliably rule out the presence of high-risk esophageal varices (HREV) in cirrhotic patients, independently of the etiology of cirrhosis [14, 15]. Therefore, the aim of this meta-analysis is to comprehensively assess the diagnostic performance of SSM for evaluating PH and EV in patients with CLD.

## Materials and methods

This study was performed in accordance with the Preferred Reporting Items for Systematic Reviews and Meta-analyses of Diagnostic Test Accuracy Studies (PRISMA-DTA) [16], and this review was registered in the International Prospective Register of Systematic Reviews (PROSPERO, http://www.crd.york.ac.uk/PROSPERO): CRD42019122407.

### Literature search

To identify studies evaluating SSM for the diagnosis of CSPH, SPH, any EV, or HREV in CLD patients, a systematic literature search was performed in PubMed, Embase, and Web of Science up to 30 April 2020. The Medical Subject Headings (MeSH) terms and free-text words terms used were as follows: spleen stiffness, portal hypertension, esophageal varices, chronic liver diseases, elastography, and diagnosis. For a comprehensive search of potentially suitable studies, a manual search was carried out by screening references of eligible articles.

### Selection criteria

Eligible studies were selected by two reviewers independently with disagreements resolved by consensus. The eligible studies were identified according to the following criteria. (1) The accuracy of SSM was evaluated for the diagnosis of CSPH, SPH, EV, or HREV in adults with CLD. (2) Portal pressure was evaluated using HVPG, and EGD was used as the reference standard for EV [17]. (3) Sufficient data was provided to calculate the true positive (TP), false positive (FP), true negative (TN), and false negative (FN) of SSM for detecting CSPH, SPH, EV, or HREV. (4) At least 30 patients were evaluated to obtain good reliability. (5) Full articles were available and written in English. Duplicate publication, animal studies, and ex vivo studies were excluded.

### Data extraction and quality assessment

Two reviewers independently extracted data and evaluated the quality of the included studies, disagreements were resolved by consensus. The following data was retrieved: first author, publication year, location, study design, technique of SSM, proportion of successful SSM, gold standard, the number of patients, age, sex, body mass index (BMI), proportion of cirrhosis, etiology of CLD, Child–Pugh score, cutoff values. TP, FP, TN, and FN were extracted directly or calculated. We limited extraction of data only to a validation cohort when both training and validation cohorts are provided in the same study. The quality of the studies was assessed according to the Quality Assessment of Diagnostic Accuracy Studies 2 tool (QUADAS-2) [18].

### Statistical analysis and data synthesis

Summary sensitivity, specificity, positive likelihood ratio (PLR), negative likelihood ratio (NLR), positive predictive value (PPV), negative predictive value (NPV), and diagnostic odds ratio (DOR) with corresponding 95% confidence intervals (CI) were calculated using the bivariate random-effects model to examine the diagnostic accuracy of SSM. Afterwards, the hierarchical summary receiver operating characteristic (HSROC) curve and the area under the curve (AUC) were calculated. Heterogeneity was evaluated using the Cochrane *Q*-test and the Higgins inconsistency index (*I*^2^), with *p* < 0.05 or *I*^2^ > 50% suggested substantial heterogeneity [19, 20]. Sensitivity analysis was performed by restricting analysis to patients with chronic viral liver disease. Univariate meta-regression analysis and subgroup analysis were also utilized to explore possible sources of heterogeneity. The covariates included the following: (1) measurement technique (MRE vs. USE), (2) study location (European vs. Asian), (3) study design (prospective vs. retrospective or cross-sectional), (4) prevalence of diseases (≥ 50% vs. < 50%), (5) proportion of cirrhosis (total vs. mixed sample), (6) etiology of CLD (viral vs. mixed), (7) proportion of Child A (≥ 50% vs. < 50%), (8) success rate of SSM (≥ 90% vs. < 90%). Fagan plots were used to assess the clinical utility of SSM for diagnosing CSPH, SPH, EV, and HREV [21]. Publication bias was assessed by Deeks’ funnel plot, with a value of *p* < 0.1 for the slope coefficient suggesting significant asymmetry [22]. All of the above analyses were performed using “midas” and “metandi” modules of Stata version 13.0 (StataCorp).

## Results

### Search results and study characteristics

**Fig. 1:**
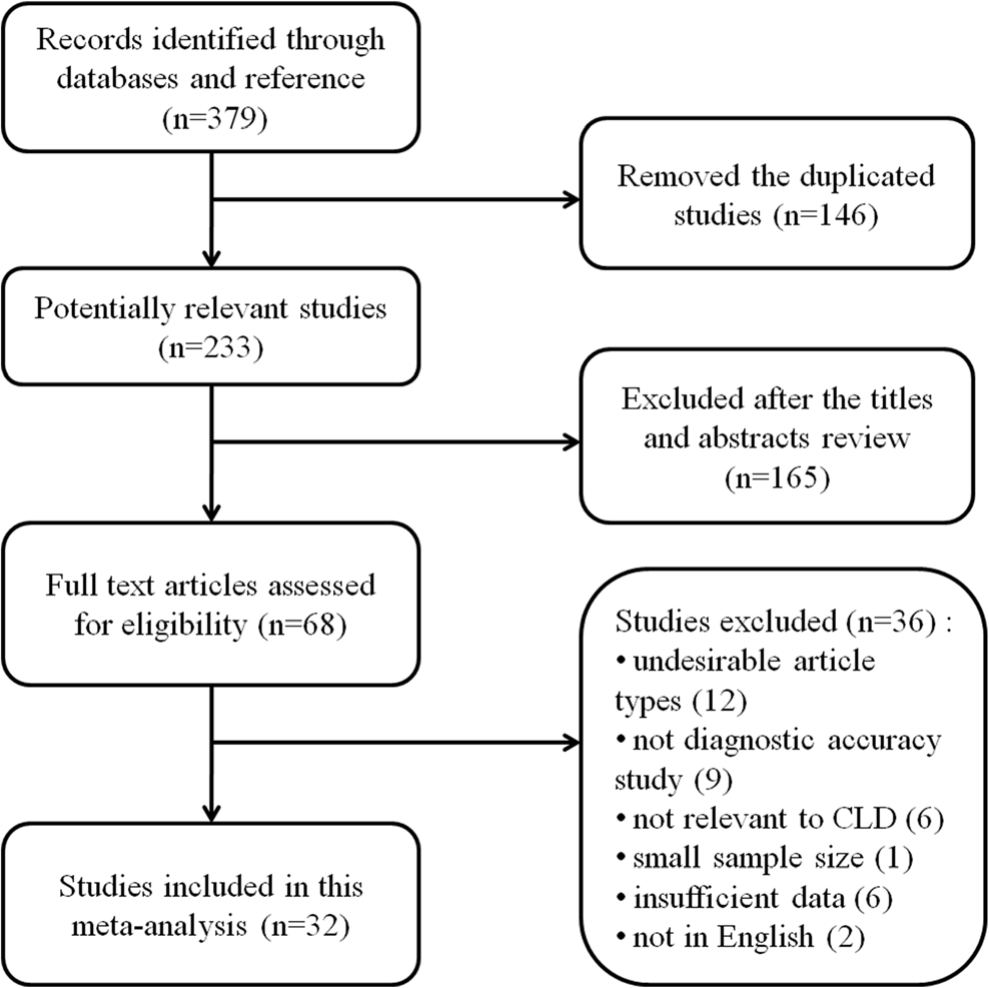
Flow chart of study selection process

The flow chart summarizing the literature screening is illustrated in Fig. 1. A total of 379 initial articles were identified with the predefined search strategies; after 146 duplicates were removed, 165 irrelevant studies were further eliminated; 68 studies were left for further evaluation. Of these, 36 articles were excluded after full-text review for the following reasons: undesirable article types, not diagnostic accuracy study, not relevant to CLD, small sample size (fewer than 30 participants), insufficient data (TP, FP, TN, and FN not reported or could not be calculated), and not in English. Ultimately, 32 articles estimating the accuracy of SSM for the diagnosis of PH and/or EV were included [11, 13–15, 23–50].

**Fig. 2:**
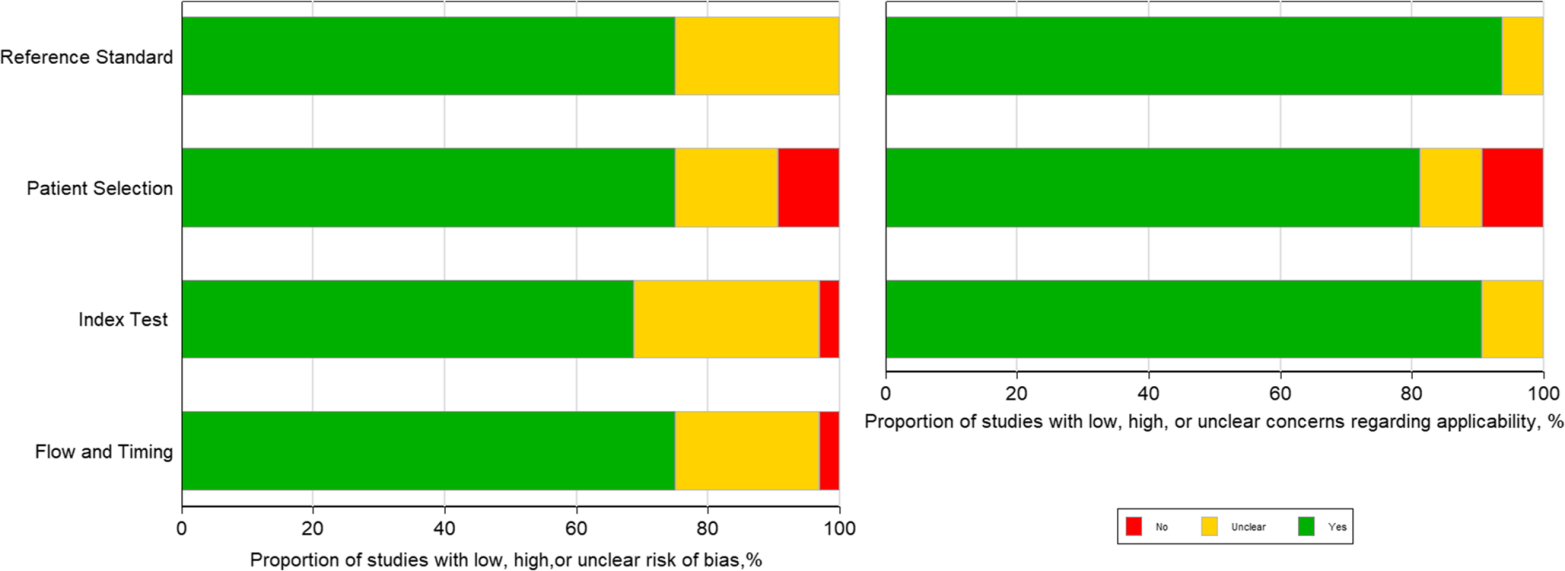
Quality assessment of the included studies according to Quality Assessment of Diagnostic Accuracy Studies-2 (QUADAS-2) criteria

According to different gold standards (HVPG and EGD), the detailed characteristics of the 32 studies were summarized in Tables 1 and 2, respectively. A total of 3952 patients with an average age of 58.8 were investigated. The 32 original articles included 15 prospective studies, 4 retrospective studies, and 13 cross-sectional studies. The results of quality assessment of the studies are shown in Fig. 2. Most studies were identified as low-risk for risk of bias and applicability concerns, with all of the studies satisfying four or more of the seven total domains (Supplementary Table 1).

**Table 1:** Characteristics of the studies evaluating the performance of spleen stiffness measurement (SSM) for the detection of portal hypertension

Author, year	Location	Study design	Technique	Manufacturer	Success rate of SSM (%)	Gold standard	No. of patients	Mean age (year)	Male (%)	Mean BMI (kg/m^2^)	Cirrhosis (%)	Etiology of CLD (viral, %)	Child–Pugh score (A/B/C)	Cutoff values-1 (CSPH)	Cutoff values-2 (SPH)
Hirooka, 2011 [23]	Japan	Prospective	RTE	Hitachi, Japan	NR	HVPG	210	62	53.8	< 25.0	NR	78.6	161/28/21	8.24	9.99
Zykus, 2015 [40]	Lithuania	Prospective	TE	FibroScan, France	92.5	HVPG	99	52	46.7	26.7	NR	63.6	69/32/1	47.60 kPa	50.70 kPa
Colecchia, 2012 [11]	Italy	Cross-sectional	TE	FibroScan, France	88.5	HVPG	100	54	71.0	25.0	100	100	68/32/0	52.80 kPa	55.00 kPa
Tseng, 2018 [45]	China	Cross-sectional	TE	FibroScan, France	90.0	HVPG	99	57	68.7	NR	100	71.2	NR	NR	48.90 kPa
Takuma, 2016 [41]	Japan	Prospective	pSWE	Siemens, Germany	96.8	HVPG	60	71	56.7	23.4	100	71.6	41/18/1	3.10 m/s	3.15 m/s
Attia, 2015 [34]	Germany	Cross-sectional	pSWE	Siemens, Germany	NR	HVPG	78	53	61.5	NR	86.0	15.0	21/46/11	2.32 m/s	2.53 m/s
Elkrief, 2015 [35]	France	Prospective	2D-SWE	Supersonic Imagine, France	97.5	HVPG	77	55	78.5	26.0	100	45.6	24/20/35	34.70 kPa	NR
Jansen, 2017 [13]	Germany	Prospective	2D-SWE	Supersonic Imagine, France	NR	HVPG	112	56	61.4	NR	100	7.6	99/45/14	26.30 kPa	28.50 kPa
Zhu, 2019 [49]	China	Prospective	2D-SWE	Supersonic Imagine, France	75.4	HVPG	104	55	62.5	20.9	100	100	65/29/10	25.30 kPa	33.40 kPa
Ronot, 2014 [32]	France	Prospective	MRE	Philips, The Netherlands	86.0	HVPG	36	56	78.0	26.0	100	42.0	7/13/16	NR	2.5 kPa

*SSM* spleen stiffness measurement, *CSPH* clinical significant portal hypertension, *SPH* severe portal hypertension, *RTE* real-time tissue elastography, *TE* transient elastography, *MRE* magnetic resonance elastography, *2D-SWE* two-dimensional shear wave elastography, *pSWE* point shear wave elastography, *HVPG* hepatic venous pressure gradient

**Table 2:** Characteristics of the studies evaluating the performance of spleen stiffness measurement (SSM) for the detection of esophageal varices

Author, year	Location	Study design	Technique	Manufacturer	The proportion of successful SSM (%)	Gold standard	No. of patients	Mean age (year)	Male (%)	Mean BMI (kg/m^2^)	Cirrhosis (%)	Etiology of CLD (viral, %)	Child–Pugh score (A/B/C)	Cutoff values-3 (EV)	Cutoff values-4 (HREV)
Hirooka, 2011 [23]	Japan	Prospectively	RTE	Hitachi, Japan	NR	EGD	210	62	53.8	< 25.0	NR	78.6	161/28/21	8.24	NR
Stefanescu, 2011 [24]	Romania	Prospectively	TE	FibroScan, France	85.4	EGD	122	56	56.2	26.4	100	NR	65/28/7	46.40 kPa	NR
Calvaruso, 2013 [28]	Italy	Prospective	TE	FibroScan, France	85.7	EGD	96	63	69.8	27.0	100	100	100/0/0	50.00 kPa	54.00 kPa^3^
Fraquelli, 2014 [30]	Italy	Prospective	TE	FibroScan, France	83.3	EGD	110	52	59.1	23.0	23.6	100	NR	65.00 kPa	NR
Colecchia, 2012 [11]	Italy	Cross-sectional	TE	FibroScan, France	88.5	EGD	100	54	71.0	25.0	100	100	68/32/0	55.00 kPa	NR
Sharma, 2013 [29]	India	Cross-sectional	TE	FibroScan, France	89.0	EGD	174	49	88.5	24.6	100	29.9	55/99/20	40.80 kPa	NR
Stefanescu, 2015 [39]	Romania	Cross-sectional	TE	FibroScan, France	NR	EGD	90	56	55.6	26.7	100	53.3	56/32/2	NR	53.00 kPa^2^
WONG, 2016 [42]	China	Cross-sectional	TE	FibroScan, France	84.1	EGD	144	58	79.2	24.4	100	100	NR	50.50 kPa	NR
Bastard, 2018 [43]	France	Cross-sectional	TE	FibroScan, France	NR	EGD	193	59	67.9	26.2	NR	NR	NR	NR	50.3 kPa^3^
Takuma, 2013 [14]	Japan	Prospectively	pSWE	Siemens, Germany	95.5	EGD	340	68	52.0	23.5	100	73.8	226/93/21	3.18 m/s	3.30 m/s^2^
Rizzo, 2014 [31]	Italy	Prospective	pSWE	Siemens, Germany	100	EGD	54	72	53.7	NR	100	100	A/B, 15/39	3.10 m/s	NR
Kim, 2015 [37]	Korea	Prospective	pSWE	Siemens, Germany	95.5	EGD	125	59	64.0	NR	100	60.8	84/32/8	3.16 m/s	3.40 m/s^3^
Takuma, 2016 [41]	Japan	Prospective	pSWE	Siemens, Germany	96.8	EGD	60	71	56.7	23.4	100	71.6	41/18/1	3.36 m/s	3.51 m/s^4^
Carmen, 2019 [47]	Romania	Prospective	pSWE	Siemens, Germany	NR	EGD	135	60	57.4	NR	100	71.1	NR	3.00 m/s	3.50 m/s^4^
Bota, 2012 [25]	Romania	Cross-sectional	pSWE	Siemens, Germany	97.9	EGD	142	59	60.0	26.7	100	50.3	66/63/16	NR	2.55 m/s^2^
Vermehren, 2012 [26]	Germany	Cross-sectional	pSWE	Siemens, Germany	100	EGD	166	54	65.7	26.0	100	48.2	A/B + C, 90/76	NR	4.13 m/s^3^
Lucchina, 2018 [44]	Italy	Cross-sectional	pSWE	Philips, The Netherlands	77.8	EGD	42	NR	NR	NR	100	61.9	NR	23.87 kPa	NR
Darweesh, 2019 [46]	Egypt	Cross-sectional	pSWE	Siemens, Germany	99.0	EGD	200	55	55.5	NR	95.5	100	A/B, 144/47	3.25 m/s	NR
Peagu, 2019 [48]	Romania	Cross-sectional	pSWE	Siemens, Germany	NR	EGD	178	60	55.1	NR	100	100	NR	2.89 m/s	3.30 m/s^5^
Giuffre, 2019 [50]	Italy	Cross-sectional	pSWE	Philips, The Netherlands	95.5	EGD	210	68	62.0	24.7	100	37.6	A/B, 179/31	31.00 kPa	46.00 kPa^5^
Ye, 2012 [27]	China	Retrospective	pSWE	Siemens, Germany	NR	EGD	73	39	59.9	21.9	100	100	NR	3.16 m/s	3.39 m/s^1^
Elkrief, 2015 [35]	France	Prospective	2D-SWE	Supersonic Imagine, France	97.5	EGD	77	55	78.5	26.0	100	45.6	24/20/35	NR	32.30 kPa^4^
Karagiannakis, 2019 [15]	Greece	Prospective	2D-SWE	Supersonic Imagine, France	90.2	EGD	64	60	50.7	NR	100	48.9	A/B, 53/18	NR	33.70 kPa^5^
Grqurevic, 2015 [36]	Croatia	Retrospective	2D-SWE	Supersonic Imagine, France	84.9	EGD	87	63	78.2	NR	100	45.6	24/20/35	30.30 kPa	NR
Ronot, 2014 [32]	France	Prospective	MRE	Philips, The Netherlands	86.0	EGD	36	56	78.0	26.0	100	42.0	7/13/16	NR	4.2 kPa^4^
Shin, 2014 [33]	South Korea	Retrospective	MRE	GE, America	96.8	EGD	139	57	73.4	NR	100	81.3	NR	7.23 kPa	7.60 kPa^3^
Morisaka, 2015 [38]	Japan	Retrospective	MRE	GE, America	NR	EGD	93	69	63.4	20.8	15.1	76.3	74/17/2	5.6 kPa	7.1 kPa^2^

*SSM* spleen stiffness measurement, *EGD* esophagogastroduodenoscopy, *EV* esophageal varices, *HREV* high-risk esophageal varices, *RTE* real-time tissue elastography, *TE* transient elastography, *MRE* magnetic resonance elastography, *2D-SWE* two-dimensional shear wave elastography, *pSWE* point shear wave elastography

^1^HREV were defined as any grade III EV

^2^HREV were defined as grade I EV with red color signs and any grade II and III EV

^3^HREV were defined as any grade II and III EV

^4^HREV were defined as any grade II and III EV or as grade I EV with red color signs or Child–Pugh class C disease

^5^HREV were defined as esophageal varices ≥ 5 mm and/or red spots and any gastric varices

### Diagnostic accuracy of SSM for the detection of CSPH

**Fig. 3:**
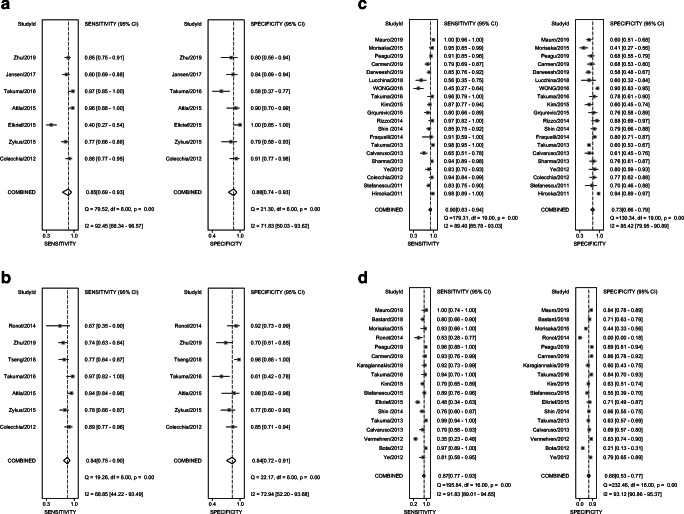
Sensitivity and specificity forest plots of spleen stiffness measurement (SSM) for detecting CSPH (**a**), SPH (**b**), EV (**c**), and HREV (**d**)

**Fig. 4:**
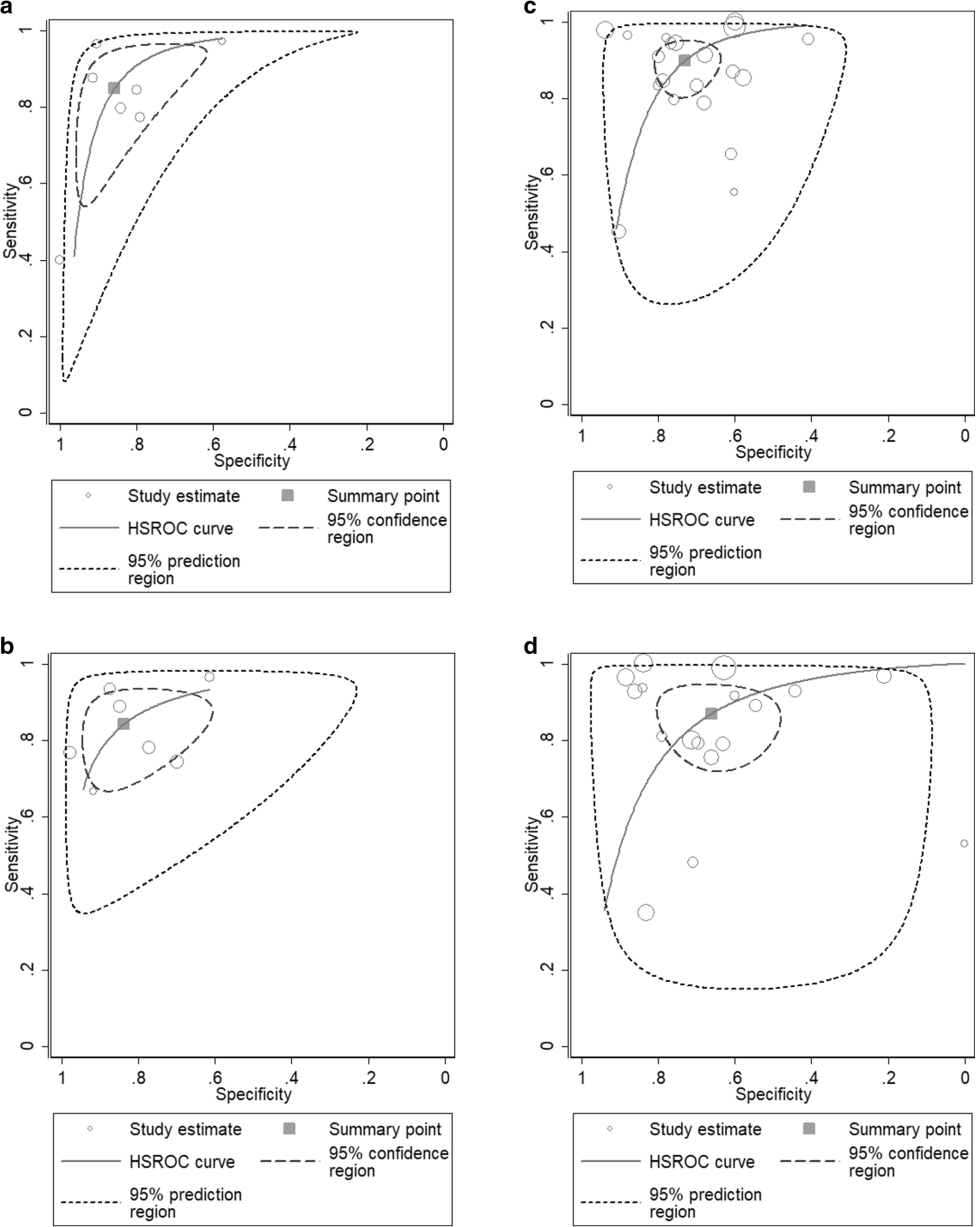
Hierarchical summary receiver operating characteristic (HSROC) curve of spleen stiffness measurement (SSM) for detecting CSPH (**a**), SPH (**b**), EV (**c**), and HREV (**d**)

The performance of SSM for the diagnosis of CSPH was evaluated in 7 studies. The pooled sensitivity and specificity of spleen stiffness for detecting CSPH were 0.85 (95% CI, 0.69–0.93) and 0.86 (95% CI, 0.74–0.93), respectively (Fig. 3a). The pooled PLR, NLR, and DOR were 5.95 (95% CI: 3.35–10.55), 0.18 (95% CI: 0.09–0.35), and 33.76 (95% CI, 16.72–68.16), respectively. Figure 4 a illustrates the HSROC curve with AUC of 0.92 (95% CI, 0.89–0.94).

### Diagnostic accuracy of SSM for the detection of SPH

The performance of SSM for the diagnosis of SPH was evaluated in 7 studies. The pooled sensitivity and specificity of SSM for detecting SPH were 0.84 (95% CI, 0.75–0.90) and 0.84 (95% CI, 0.72–0.91), respectively (Fig. 3b). The pooled PLR, NLR, and DOR were 5.17 (95% CI: 2.94–9.10), 0.19 (95% CI: 0.12–0.30), and 27.47 (95% CI, 12.79–59.00), respectively. Figure 4b illustrates the HSROC curve with AUC of 0.91 (95% CI, 0.88–0.93).

### Diagnostic accuracy of SSM for the detection of any EV

The diagnostic accuracy of SSM for EV was evaluated in 20 studies. The pooled sensitivity and specificity of SSM for detecting CSPH were 0.90 (95% CI, 0.83–0.94) and 0.73 (95% CI, 0.66–0.79), respectively (Fig. 3c). The pooled PLR, NLR, and DOR were 3.34 (95% CI: 2.63–4.24), 0.14 (95% CI: 0.08–0.23), and 23.84 (95% CI, 12.70–44.74), respectively. Figure 4c illustrates the HSROC curve with AUC of 0.87 (95% CI, 0.84–0.90). On restricting analysis to 8 studies performed in pure chronic viral liver disease, the pooled sensitivity and specificity was 0.85 (95% CI, 0.72–0.92) and 0.76 (95% CI, 0.67–0.84), with an AUC of 0.86 (95% CI, 0.83–0.89). The sensitivity analysis did not significantly increase the diagnostic performance of SSM.

### Diagnostic accuracy of SSM for the detection of HREV

The diagnostic accuracy of SSM for HREV was evaluated in 17 studies. HREV were variably defined in the included studies (Table 2). The pooled sensitivity and specificity of SSM for detecting HREV were 0.87 (95% CI, 0.77–0.93) and 0.66 (95% CI, 0.53–0.77), respectively (Fig. 4c). The pooled PLR, NLR, and DOR were 2.56 (95% CI: 1.76–3.72), 0.20 (95% CI: 0.10–0.38), and 13.01 (95% CI, 5.19–32.64), respectively. Figure 4d illustrates the HSROC curve with AUC of 0.83 (95% CI, 0.79–0.86). On the basis of these values, and assuming a 29.9% HREV (as observed in the included studies), the pooled PPV and NPV were 0.54 (95% CI: 0.47–0.62) and 0.88 (95% CI: 0.81–0.95), respectively. Considering the pooled NPV and the prevalence of HREV in the included studies, a total of 50.6% (95% CI, 43.4–59.0%) patients would avoid endoscopies with a risk of missing HREV of 8.4% (95% CI, 4.1–17.2%) in patients with the “negative” results of SSM, and 4.7% (95% CI, 2.3–9.4%) among the overall population of 2214 patients evaluated (Table 3).

**Table 3:** Summary diagnostic accuracy and the post-test probabilities of spleen stiffness measurement (SSM) for CSPH, SPH, EV, and HREV

	No. of studies	Sensitivity (95% CI)	Specificity (95% CI)	PPV (95% CI)	NPV (95% CI)	PLR (95% CI)	NLR (95% CI)	DOR (95% CI)	*I*^2^	AUC (95% CI)	Pre-test probability (%)	Post-test probability (+) (%)	Post-test probability (−) (%)
CSPH	7	0.85 (0.69–0.93)	0.86 (0.74–0.93)	0.94 (0.90–0.98)	0.68 (0.54–0.84)	5.95 (3.35–10.55)	0.18 (0.09–0.35)	33.76 (16.72–68.16)	97.24%	0.92 (0.89–0.94)	25	66	6
											50	86	15
											75	95	35
SPH	7	0.84 (0.75–0.90)	0.84 (0.72–0.91)	0.89 (0.82–0.96)	0.78 (0.67–0.90)	5.17 (2.94–9.10)	0.19 (0.12–0.30)	27.47 (12.79–59.00)	99.34%	0.91 (0.88–0.93)	25	63	6
											50	84	16
											75	94	36
EV	20	0.90 (0.83–0.94)	0.73 (0.66–0.79)	0.76 (0.69–0.83)	0.81 (0.74–0.89)	3.34 (2.63–4.24)	0.14 (0.08–0.23)	23.84 (12.70–44.74)	100.00%	0.87 (0.84–0.90)	25	53	4
											50	77	12
											75	91	30
HREV	17	0.87 (0.77–0.93)	0.66 (0.53–0.77)	0.54 (0.47–0.62)	0.88 (0.81–0.95)	2.56 (1.76–3.72)	0.20 (0.10–0.38)	13.01 (5.19–32.64)	100.00%	0.83 (0.79–0.86)	25	46	6
											50	72	16
											75	88	37

*SSM* spleen stiffness measurement, *CSPH* clinical significant portal hypertension (HVPG ≥ 10 mmHg), *SPH* severe portal hypertension (HVPG ≥ 12 mmHg), *EV* esophageal varices, *HREV* high-risk esophageal varices, *PPV* positive predictive value, *NPV* negative predictive value, *PLR* positive likelihood ratio, *NLR* negative likelihood ratio, *DOR* diagnostic odds ratio, *AUC* area under the curve

Significant heterogeneity among studies was observed in DOR (*p* < 0.001). The Deeks’ plot showed that there was no potential publication bias for the studies (*p* = 0.60, 0.95, 0.15, 0.14) (Supplementary Fig. 1).

### Results of meta-regression and subgroup analysis

Univariate meta-regressions showed that the types of elastography technique, study location, study design, prevalence of diseases, etiology of CLD, proportion of Child A, and success rate of SSM were associated with the heterogeneity. SSM showed better performance for the diagnosis of any EV in Asian populations than in European populations. In addition, compared with the studies having a success rate of SSM < 90%, studies with a success rate ≥ 90% had a lower specificity for the diagnosis of any EV. The details of subgroup analysis are demonstrated in Table 4.

**Table 4:** Results of subgroup analysis of spleen stiffness measurement (SSM) for the diagnosis of CSPH, SPH, EV, and HREV

Covariates	Subgroup	CSPH		SPH		EV		HREV	
		Sensitivity	Specificity	Sensitivity	Specificity	Sensitivity	Specificity	Sensitivity	Specificity
Technique	1-MRE	/	/	0.67 (0.32–1.00)	0.92 (0.78–1.00)	0.92 (0.78–1.00)	0.61 (0.39–0.84)	0.77 (0.50–1.00)	0.34 (0.08–0.61)*
	0-USE	/	/	0.85 (0.78–0.92)	0.82 (0.72–0.92)	0.90 (0.84–0.95)	0.74 (0.68–0.81)	0.88 (0.81–0.96)	0.72 (0.62–0.82)*
Location	1-European	0.80 (0.65–0.95)	0.89 (0.83–0.95)	0.85 (0.76–0.94)	0.86 (0.75–0.97)	0.88 (0.80–0.97)^*^	0.72 (0.62–0.81)*	0.85 (0.74–0.95)	0.65 (0.50–0.80)
	0-Asian	0.93 (0.82–1.00)	0.69 (0.51–0.86)	0.83 (0.71–0.95)	0.81 (0.65–0.96)	0.92 (0.85–0.98)*	0.76 (0.68–0.85)*	0.90 (0.81–1.00)	0.68 (0.48–0.87)
Design	1-Prospective	0.79 (0.65–0.94)	0.83 (0.71–0.95)	0.79 (0.68–0.90)*	0.76 (0.64–0.88)**	0.92 (0.85–0.98)	0.75 (0.67–0.84)	0.86 (0.74–0.98)	0.63 (0.45–0.82)
	0-Retrospective or cross-sectional	0.93 (0.83–1.00)	0.91 (0.79–1.00)	0.88 (0.79–0.96)^*^	0.91 (0.83–0.98)**	0.88 (0.81–0.96)	0.71 (0.62–0.80)	0.88 (0.78–0.98)	0.53 (0.53–0.84)
Prevalence	1–≥ 50%	/	/	0.83 (0.75–0.92)	0.85 (0.75–0.95)	0.86 (0.78–0.94)**	0.73 (0.64–0.81)*	0.73 (0.38–1.00)	0.63 (0.26–1.00)
	0–< 50%	/	/	0.87 (0.73–1.00)	0.79 (0.59–0.99)	0.94 (0.90–0.99)**	0.73 (0.64–0.83)*	0.88(0.81–0.96)	0.66 (0.54–0.79)
Cirrhosis	1-total	0.82 (0.68–0.96)	0.87 (0.75–0.99)	0.82 (0.74–0.91)*	0.85 (0.73–0.96)	0.88 (0.82–0.94)	0.73 (0.67–0.79)	0.87 (0.79–0.96)	0.67 (0.54–0.80)
	0-mixed	0.97 (0.89–1.00)	0.91 (0.71–1.00)	0.94 (0.86–1.00)*	0.89 (0.66–1.00)	0.91 (0.79–1.00)	0.61 (0.47–0.75)	0.95 (0.77–1.00)	0.44 (0.10–0.98)
Etiology (% viral)	1-viral	0.86 (0.68–1.00)	0.87 (0.73–1.00)	0.82 (0.68–0.96)	0.79 (0.59–0.98)	0.86 (0.75–0.96)*	0.76 (0.67–0.86)*	0.89 (0.73–1.00)	0.80 (0.61–1.00)
	0-mixed	0.84 (0.70–0.98)	0.85 (0.73–0.96)	0.85 (0.77–0.94)	0.86 (0.75–0.96)	0.93 (0.87–0.98)*	0.71 (0.62–0.80)*	0.87 (0.78–0.96)	0.61 (0.47–0.76)
Child A (%)	1–≥ 50%	0.87 (0.75–0.99)	0.80 (0.68–0.93)	0.85 (0.75–0.95)	0.75 (0.66–0.83)*	0.94 (0.89–0.99)	0.68 (0.58–0.78)*	0.90 (0.79–1.00)	0.70 (0.57–0.83)*
	0–< 50%	0.79 (0.51–1.00)	0.97 (0.92–1.00)	0.87 (0.72–1.00)	0.90 (0.80–1.00)*	0.92 (0.82–1.00)	0.80 (0.66–0.95)*	0.75 (0.43–1.00)	0.26 (0.04–0.48)*
Successful rate of SSM (%)	1–≥ 90%	0.76 (0.55–0.98)	0.85 (0.67–1.00)	0.84 (0.74–0.94)	0.83 (0.68–0.98)	0.95 (0.90–0.99)	0.68 (0.60–0.76)***	0.88 (0.76–0.99)	0.68 (0.51–0.84)
	0–< 90%	0.86 (0.69–1.00)	0.88 (0.70–1.00)	0.79 (0.67–0.90)	0.84 (0.69–0.99)	0.80 (0.68–0.93)	0.77 (0.70–0.83)***	0.68 (0.22–1.00)	0.26 (–0.08–0.63)

*SSM* spleen stiffness measurement, *CSPH* clinical significant portal hypertension (HVPG ≥ 10 mmHg), *SPH* severe portal hypertension (HVPG ≥ 12 mmHg), *EV* esophageal varices, *HREV* high-risk esophageal varices, *TE* transient elastography, *SWE* shear wave elastography

*There were significant differences between two subgroups (*p* < 0.05)

**There were significant differences between two subgroups (*p* < 0.01)

***There were significant differences between two subgroups (*p* < 0.001)

### Clinical utility of SSM for detecting CSPH, SPH, EV, and HREV

The Fagan plot analysis indicated that when pre-test probability was 50%, SSM was very informative with an 86% probability of correctly detecting CSPH following a “positive” measurement and lowering the probability of disease to 15% when “negative” measurement; and the probability of correctly diagnosing SPH following a “positive” measurement reached 84%. However, the probability of a correct diagnosis rate did not exceed 80% for diagnosing any EV and HREV when the pre-test probability was 50% (Table 3).

## Discussion

The results of this meta-analysis indicated that spleen stiffness measured by current techniques had a fairly good accuracy for the detection of PH and EV in CLD patients. AUCs for the diagnosis of CSPH and SPH exceeded 90%, and AUCs for diagnosis of any EV and HREV reached 87% and 83%, respectively. SSM was able to predict the presence of CSPH with good sensitivity and specificity (85% and 86%, respectively). Notably, we observed that the pooled sensitivity and NPV of SSM for detecting HREV were fairly good, and was 0.87 (95% CI, 0.77–0.93) and 0.88 (95% CI, 0.81–0.95), respectively, which suggested that HREV could be ruled out in most CLD patients evaluated by SSM, thereby avoiding unnecessary endoscopy.

PH results in progressive splenomegaly and remodeled spleen, which, due to passive congestion, increased arterial blood flow and fibrogenesis that may enhance spleen stiffness, lending support to the physiological feasibility of SSM for detecting PH and EV [51, 52]. Previous studies have confirmed that USE showed good diagnostic performance for significant liver fibrosis and liver cirrhosis [53, 54]. MRE is a newly developed method to quantitatively evaluate the elasticity of living tissue that provides full-field-of-view elastograms of the abdomen with excellent diagnostic accuracy for staging hepatic fibrosis [55, 56]. Studies have demonstrated that MRE-based spleen stiffness is strongly associated with the presence of EV, and with the cutoff value of 7.23 kPa, SSM showed good performance for detecting EV in cirrhosis patients, with an AUC of 0.83 (95% CI, 0.76–0.89) [33, 38]. In the past several years, MRE-based spleen stiffness has been suggested as a valid parameter to identify the presence of EV [57].

The prevalence of varices needing treatment (VNT) is very low in patients with compensated cirrhosis [58]. Previous studies suggest that liver stiffness measurement (LSM) plus platelet count can be used to exclude the presence of HREV in patients with Child–Pugh A cirrhosis [59]. However, the performance of LSM alone in predicting PH is controversial due to lack of consistent results, which may be due to the reason that it is affected by confounding factors, such as hepatocyte inflammation and cholestasis, and it only reflects the increase of intrahepatic resistance to portal blood flow, while is unable to account for dynamic changes of the splanchnic blood flow [8]. In a meta-analysis focusing on the diagnostic performance of LSM, the DOR for evaluating any EV and HREV was 7.54 (95% CI, 4.46–12.73) and 8.85 (95% CI, 5.93–13.19), respectively [60]. In our meta-analysis, the comparable DOR of SSM were 21.92 (95% CI, 11.53-41.68) and 16.07 (95% CI, 7.15-36.14), respectively. The results show that the diagnostic accuracy of SSM for detecting EV was significantly better than that of LSM. Considering the pooled NPV (0.88) and the prevalence of HREV observed in the included studies (29.9%), a total of 1120 (50.6%) patients would avoid endoscopies with a risk of missing HREV of 4.7% among the overall 2214 patients evaluated. As compared with the Expanded-Baveno VI criteria, SSM would spare more unnecessary endoscopies (50.6% vs. 40.0%); however, the number of HREV missed increased as well (4.7% vs. 1.6%) [61]. The increase of missed diagnosis rate may be due to the prevalence rate of HREV, which is significantly greater in our meta-analysis than in the cohort of the Expanded-Baveno VI criteria (29.9% vs. 9.9%), and the NPV is affected by the prevalence of disease. When the prevalence rate is high, the NPV is relatively low, resulting in an increased rate of missed diagnosis. Accordingly, our meta-analysis demonstrated that SSM was useful for ruling out the presence of HREV in CLD patients, and a new model combined with SSM and other noninvasive criteria would probably safely avoid more endoscopies [62].

Considerable heterogeneity was observed in our study and a meta-regression analysis was performed to identify probable causes. We observed that the diagnostic performance of SSM for detecting any EV was better across Asian populations than in European populations. Previous studies have shown that BMI and central obesity are independent influencing factors for the failure and unreliability of USE [63]. The mean BMI of the subjects from European was higher (range: 23.0–27.0 kg/m^2^) than that of Asian subjects (range: 20.8–24.6 kg/m^2^). In addition, compared with the studies with a success rate of SSM < 90%, the studies with a success rate ≥ 90% had a lower specificity for detecting any EV. This may be due to the thickness of spleen, which may have affected the success rate of SSM, and when the thickness of the spleen was less than 4 cm, the success rate of SSM was low. Furthermore, the prevalence of EV increases with the degree of splenomegaly, which would lead to a decrease in the specificity of the detection.

The main strength of our study is that we comprehensively evaluated the diagnostic accuracy of spleen stiffness, measured by different techniques including USE and MRE, across variety of populations and chronic liver disease. Therefore, the result of our meta-analysis would reflect the diagnostic performance of SSM for detecting PH and EV in a real world. In addition, we separately assessed the diagnostic accuracy of SSM in detecting CSPH, SPH, any EV, and HREV, in order to evaluate the clinical application value of SSM comprehensively.

There were several limitations in this study. First, a considerable amount of heterogeneity was detected across the included studies, attributable to the types of elastography technique, study location, study design, the prevalence of disease, and several other covariates which were unrecorded in the included studies. Second, the number of eligible studies was relatively low, with only 3 studies having assessed MRE, and some relatively small samples of studies were included in our meta-analysis. In the future, large-sample and multicenter studies are needed for more comprehensive evaluation. In addition, our meta-analysis included only studies written in English, putting the results at risk of language bias. Considering these limitations, caution must be taken when interpreting the results of our study.

In conclusion, SSM was a promising method to detecting PH and EV with good diagnostic accuracy and it would be a helpful noninvasive surveillance tool for clinicians in management CLD patients. In addition, SSM could rule out the presence of HREV in most CLD patients and would be used as an initial screening method thereby avoiding unnecessary endoscopy. Future, prospective studies with larger sample size and in diverse clinical settings are required to further assess the effectiveness of SSM.

## Electronic supplementary material

ESM 1 (DOCX 12391 kb)

## Abbreviations

CLD: Chronic liver diseases
CSPH: Clinical significant portal hypertension
EGD: Esophagogastroduodenoscopy
EV: Esophageal varices
HREV: High-risk esophageal varices
HVPG: Hepatic venous pressure gradient
LSM: Liver stiffness measurement
MRE: Magnetic resonance elastography
PH: Portal hypertension
RTE: Real-time tissue elastography
SPH: Severe portal hypertension
SSM: Spleen stiffness measurement
SWE: Shear wave elastography
TE: Transient elastography
USE: Ultrasound elastography
VNT: Varices needing treatment
