# Correction to: Diagnostic accuracy of spleen stiffness to evaluate portal hypertension and esophageal varices in chronic liver disease: a systematic review and meta-analysis

**DOI:** 10.1007/s00330-020-07446-9

**Published:** 2020-11-18

**Authors:** Xing Hu, Xiaojie Huang, Jianhua Hou, Lei Ding, Chunling Su, Fankun Meng

**Affiliations:** 1 Ultrasound and Functional Diagnosis Center, Beijing Youan Hospital, Capital Medical University, No. 8, Xitoutiao, Youanmenwai, Fengtai District, Beijing, 100069 China; 2 Center for Infectious Disease, Beijing Youan Hospital, Capital Medical University, Beijing, China

**Correction to: European Radiology**

10.1007/s00330-020-07223-8

The original version of this article, published on 24 September 2020, unfortunately contained a mistake. Reference 47 was incorrectly cited in Table 2 and Fig. 3. The corrected versions are given below.

**Table 2:** Characteristics of the studies evaluating the performance of spleen stiffness measurement (SSM) for the detection of esophageal varices

Author, year	Location	Study design	Technique	Manufacturer	The proportion of successful SSM (%)	Gold standard	No. of patients	Mean age (year)	Male (%)	Mean BMI (kg/m^2^)	Cirrhosis (%)	Etiology of CLD (viral, %)	Child–Pugh score (A/B/C)	Cutoff values-3 (EV)	Cutoff values-4 (HREV)
Hirooka, 2011 [23]	Japan	Prospectively	RTE	Hitachi, Japan	NR	EGD	210	62	53.8	< 25.0	NR	78.6	161/28/21	8.24	NR
Stefanescu, 2011 [24]	Romania	Prospectively	TE	FibroScan, France	85.4	EGD	122	56	56.2	26.4	100	NR	65/28/7	46.40 kPa	NR
Calvaruso, 2013 [28]	Italy	Prospective	TE	FibroScan, France	85.7	EGD	96	63	69.8	27.0	100	100	100/0/0	50.00 kPa	54.00 kPa^3^
Fraquelli, 2014 [30]	Italy	Prospective	TE	FibroScan, France	83.3	EGD	110	52	59.1	23.0	23.6	100	NR	65.00 kPa	NR
Colecchia, 2012 [11]	Italy	Cross-sectional	TE	FibroScan, France	88.5	EGD	100	54	71.0	25.0	100	100	68/32/0	55.00 kPa	NR
Sharma, 2013 [29]	India	Cross-sectional	TE	FibroScan, France	89.0	EGD	174	49	88.5	24.6	100	29.9	55/99/20	40.80 kPa	NR
Stefanescu, 2015 [39]	Romania	Cross-sectional	TE	FibroScan, France	NR	EGD	90	56	55.6	26.7	100	53.3	56/32/2	NR	53.00 kPa^2^
WONG, 2016 [42]	China	Cross-sectional	TE	FibroScan, France	84.1	EGD	144	58	79.2	24.4	100	100	NR	50.50 kPa	NR
Bastard, 2018 [43]	France	Cross-sectional	TE	FibroScan, France	NR	EGD	193	59	67.9	26.2	NR	NR	NR	NR	50.3 kPa^3^
Takuma, 2013 [14]	Japan	Prospectively	pSWE	Siemens, Germany	95.5	EGD	340	68	52.0	23.5	100	73.8	226/93/21	3.18 m/s	3.30 m/s^2^
Rizzo, 2014 [31]	Italy	Prospective	pSWE	Siemens, Germany	100	EGD	54	72	53.7	NR	100	100	A/B, 15/39	3.10 m/s	NR
Kim, 2015 [37]	Korea	Prospective	pSWE	Siemens, Germany	95.5	EGD	125	59	64.0	NR	100	60.8	84/32/8	3.16 m/s	3.40 m/s^3^
Takuma, 2016 [41]	Japan	Prospective	pSWE	Siemens, Germany	96.8	EGD	60	71	56.7	23.4	100	71.6	41/18/1	3.36 m/s	3.51 m/s^4^
Fierbinteanu-Braticevici, 2019 [47]	Romania	Prospective	pSWE	Siemens, Germany	NR	EGD	135	60	57.4	NR	100	71.1	NR	3.00 m/s	3.50 m/s^4^
Bota, 2012 [25]	Romania	Cross-sectional	pSWE	Siemens, Germany	97.9	EGD	142	59	60.0	26.7	100	50.3	66/63/16	NR	2.55 m/s^2^
Vermehren, 2012 [26]	Germany	Cross-sectional	pSWE	Siemens, Germany	100	EGD	166	54	65.7	26.0	100	48.2	A/B + C, 90/76	NR	4.13 m/s^3^
Lucchina, 2018 [44]	Italy	Cross-sectional	pSWE	Philips, Netherlands	77.8	EGD	42	NR	NR	NR	100	61.9	NR	23.87 kPa	NR
Darweesh, 2019 [46]	Egypt	Cross-sectional	pSWE	Siemens, Germany	99.0	EGD	200	55	55.5	NR	95.5	100	A/B, 144/47	3.25 m/s	NR
Peagu, 2019 [48]	Romania	Cross-sectional	pSWE	Siemens, Germany	NR	EGD	178	60	55.1	NR	100	100	NR	2.89 m/s	3.30 m/s^5^
Giuffre, 2019 [50]	Italy	Cross-sectional	pSWE	Philips, Netherlands	95.5	EGD	210	68	62.0	24.7	100	37.6	A/B, 179/31	31.00 kPa	46.00 kPa^5^
Ye, 2012 [27]	China	Retrospective	pSWE	Siemens, Germany	NR	EGD	73	39	59.9	21.9	100	100	NR	3.16 m/s	3.39 m/s^1^
Elkrief, 2015 [35]	France	Prospective	2D-SWE	Supersonic Imagine, France	97.5	EGD	77	55	78.5	26.0	100	45.6	24/20/35	NR	32.30 kPa^4^
Karagiannakis, 2019 [15]	Greece	Prospective	2D-SWE	Supersonic Imagine, France	90.2	EGD	64	60	50.7	NR	100	48.9	A/B, 53/18	NR	33.70 kPa^5^
Grqurevic, 2015 [36]	Croatia	Retrospective	2D-SWE	Supersonic Imagine, France	84.9	EGD	87	63	78.2	NR	100	45.6	24/20/35	30.30 kPa	NR
Ronot, 2014 [32]	France	Prospective	MRE	Philips, Netherlands	86.0	EGD	36	56	78.0	26.0	100	42.0	7/13/16	NR	4.2 kPa^4^
Shin, 2014 [33]	South Korea	Retrospective	MRE	GE, America	96.8	EGD	139	57	73.4	NR	100	81.3	NR	7.23 kPa	7.60 kPa^3^
Morisaka, 2015 [38]	Japan	Retrospective	MRE	GE, America	NR	EGD	93	69	63.4	20.8	15.1	76.3	74/17/2	5.6 kPa	7.1 kPa^2^

*SSM* spleen stiffness measurement, *EGD* esophagogastroduodenoscopy, *EV* esophageal varices, *HREV* high-risk esophageal varices, *RTE* real-time tissue elastography, *TE* transient elastography, *MRE* magnetic resonance elastography, *2D-SWE* two-dimensional shear wave elastography, *pSWE* point shear wave elastography

^1^HREV were defined as any grade III EV

^2^HREV were defined as grade I EV with red color signs and any grade II and III EV

^3^HREV were defined as any grade II and III EV

^4^HREV were defined as any grade II and III EV or as grade I EV with red color signs or Child–Pugh class C disease

^5^HREV were defined as esophageal varices ≥ 5 mm and/or red spots and any gastric varices

**Fig. 3:**
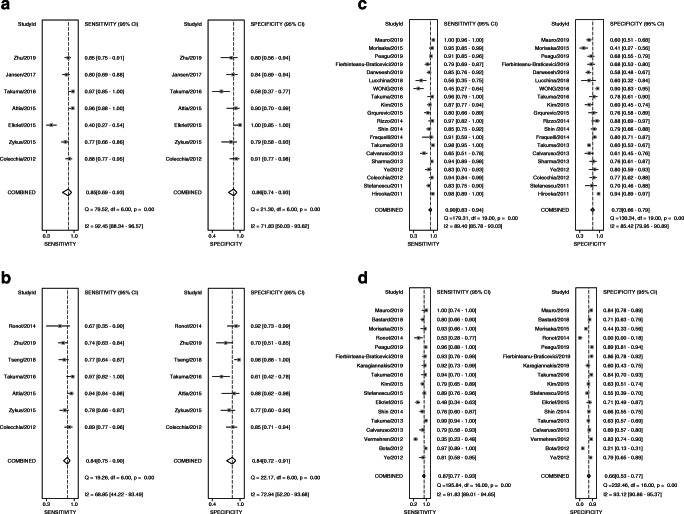
Sensitivity and specificity forest plots of spleen stiffness measurement (SSM) for detecting CSPH (**a**), SPH (**b**), EV (**c**), and HREV (**d**)

