# Supplementary material for: Diagnostic accuracy of spleen stiffness to evaluate portal hypertension and esophageal varices in chronic liver disease: a systematic review and meta-analysis
Source: Eur Radiol. 2020 Sep 24;31(4):2392–404. doi: 10.1007/s00330-020-07223-8 (PMC7979650; doi:10.1007/s00330-020-07223-8)

**Supplemental Materials**

**Supplementary Table 1 Quality assessment with QUADAS-2.**

| Reference | Risk of bias | | | | Applicability concerns | | |
| --- | --- | --- | --- | --- | --- | --- | --- |
|  | Patient selection | Index test | Reference standard | Flow and timing | Patient selection | Index test | Reference standard |
| Hirooka, 2011 | Low | Low | Low | Low | Low | Low | Low |
| Stefanescu,2011 | Low | Unclear | Low | Unclear | Low | Low | Low |
| Colecchia, 2012 | Low | Low | Low | Low | Low | Low | Low |
| Ye, 2012 | High | High | Low | Unclear | High | Low | Low |
| Bota, 2012 | Low | Low | Low | Low | Low | Low | Low |
| Vermehren, 2012 | Low | Unclear | Unclear | Low | Low | Low | Low |
| Sharma, 2013 | Low | Low | Low | Low | Low | Low | Low |
| Calvaruso, 2013 | Low | Unclear | Low | Unclear | Low | Low | Low |
| Takuma, 2013 | Low | Low | Low | Low | Low | Low | Low |
| Fraquelli, 2014 | Unclear | Low | Low | Unclear | Low | Low | Low |
| Shin, 2014 | Unclear | Low | Low | Low | Unclear | Low | Low |
| Ronot, 2014 | Low | Low | Low | Low | Low | Low | Low |
| Rizzo, 2014 | Unclear | Low | Low | Low | Unclear | Low | Low |
| Morisaka, 2015 | Low | Low | Low | Low | Low | Low | Low |
| Grqurevic, 2015 | High | Low | Unclear | Unclear | High | Low | Low |
| Zykus, 2015 | Unclear | Low | Low | Low | Low | Low | Low |
| Elkrief, 2015 | Low | Unclear | Low | Low | Low | Unclear | Low |
| Stefanescu, 2015 | Low | Low | Low | Low | Low | Low | Low |
| Attia, 2015 | Low | Low | Low | Low | Low | Low | Low |
| Kim, 2015 | Low | Low | Unclear | Low | Low | Unclear | Unclear |
| Takuma, 2016 | Low | Low | Low | Low | Low | Low | Low |
| WONG, 2016 | Low | Low | Low | Low | Low | Low | Low |
| Jansen, 2017 | Low | Unclear | Low | Low | Low | Unclear | Low |
| Tseng, 2018 | High | Low | Unclear | Low | High | Low | Unclear |
| Lucchina, 2018 | Unclear | Low | Low | High | Unclear | Low | Low |
| Bastard, 2018 | Low | Unclear | Unclear | Unclear | Low | Low | Low |
| Zhu, 2019 | Low | Low | Low | Low | Low | Low | Low |
| Karagiannakis, 2019 | Low | Low | Low | Low | Low | Low | Low |
| Darweesh, 2019 | Low | Unclear | Unclear | Low | Low | Low | Low |
| Carmen, 2019 | Low | Unclear | Unclear | Low | Low | Low | Low |
| Peagu, 2019 | Low | Low | Low | Low | Low | Low | Low |
| Mauro, 2019 | Low | Unclear | Unclear | Unclear | Low | Low | Low |

QUADAS-2: Quality assessment of diagnostic accuracy studies-2; Low: Low Risk; High: High Risk; Unclear: Unclear Risk

**Supplementary Figure 1 Deeks’ funnel plot asymmetry test for publication bias.**

1. CSPH; (b) SPH; (c) EV; (d)HREV

**
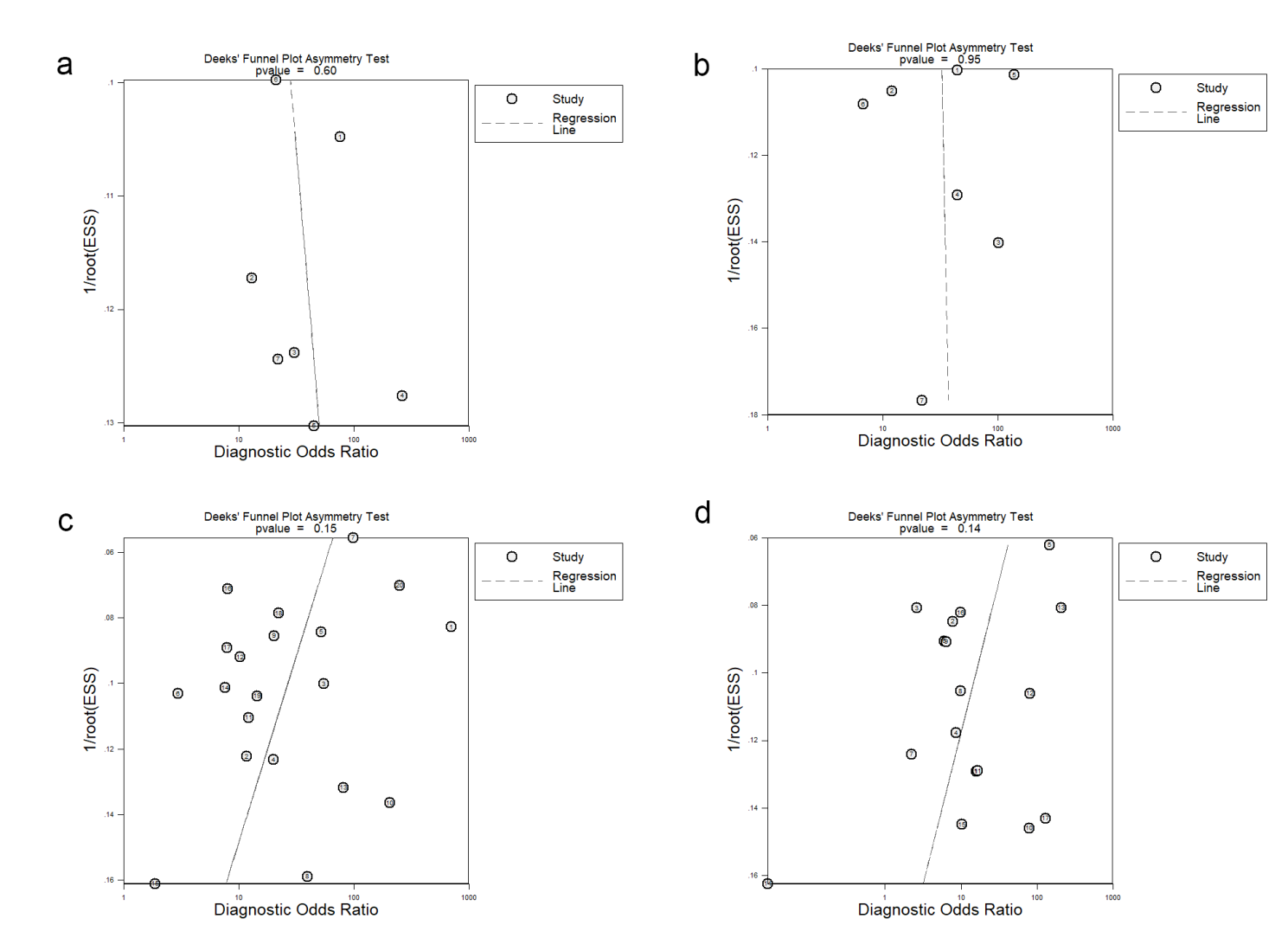
**

Deeks’ funnel plot indicate that no signifcant bias was found (*P*=0.60, 0.95, 0.15, 0.14).

**Supplementary Figure 2 Fagan plot to evaluate the clinical utility of SSM for detecting CSPH.**

(a) Pre-test probability=25%; (b) Pre-test probability=50%; (c) Pre-test probability=75%.

NLR: negative likelihood ratio; PLR: positive likelihood ratio


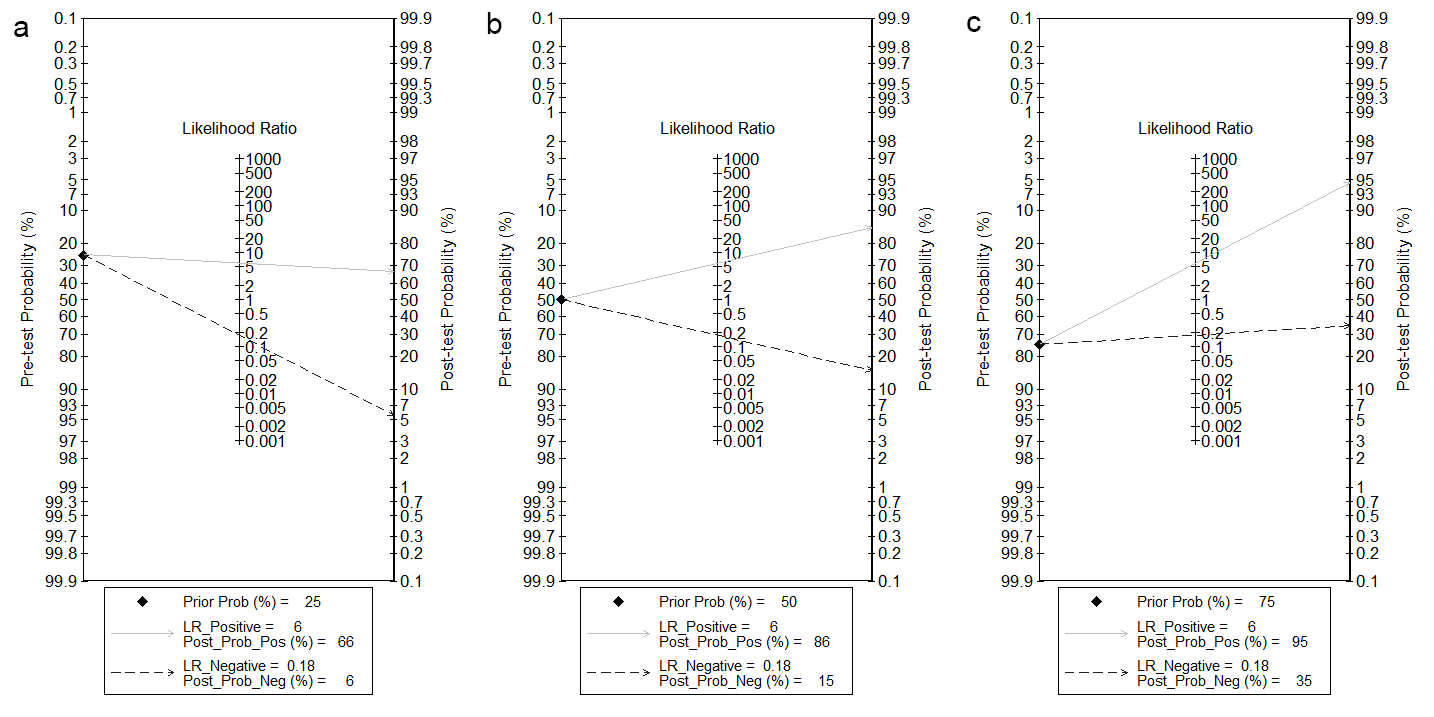


**Supplementary Figure 3 Fagan plot to evaluate the clinical utility of SSM for detecting SPH.**

(a) Pre-test probability=25%; (b) Pre-test probability=50%; (c) Pre-test probability=75%.

NLR: negative likelihood ratio; PLR: positive likelihood ratio


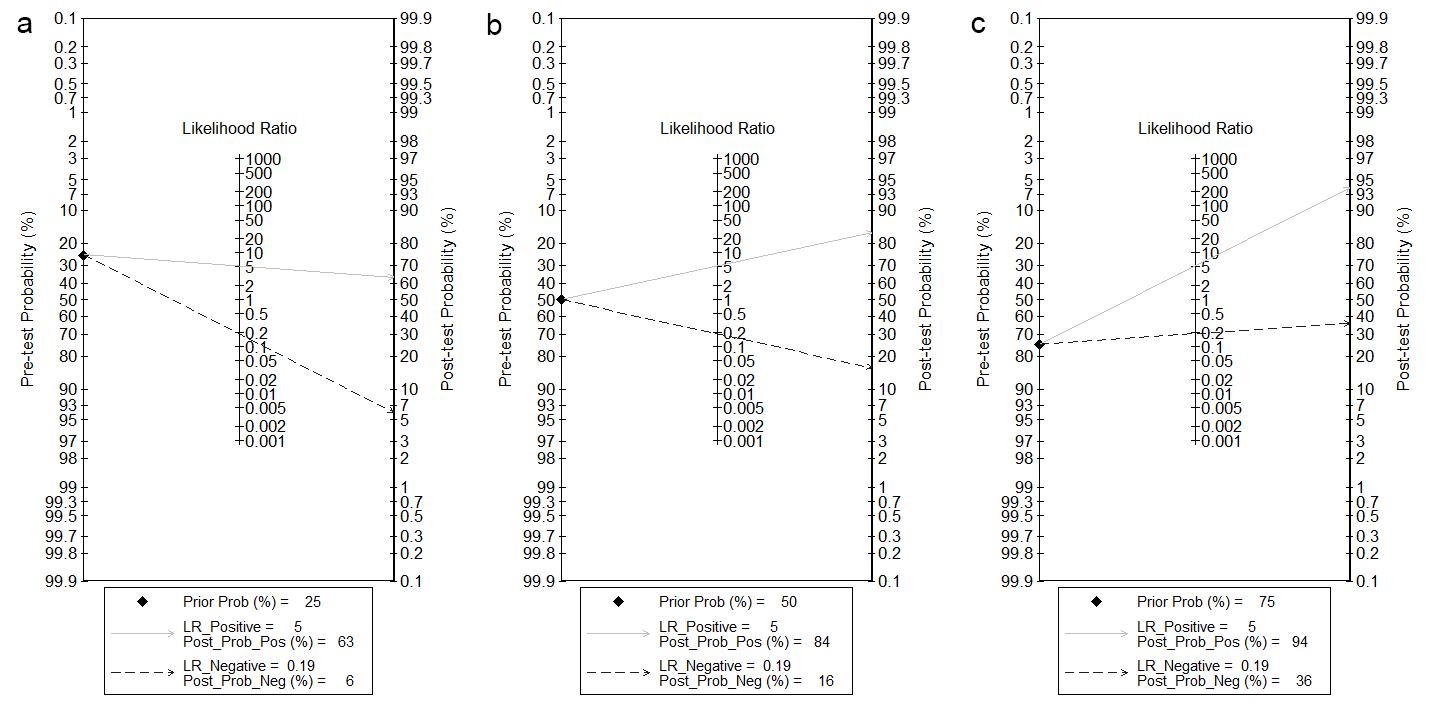


**Supplementary Figure 4 Fagan plot to evaluate the clinical utility of SSM for detecting EV.**

(a) Pre-test probability=25%; (b) Pre-test probability=50%; (c) Pre-test probability=75%.

NLR: negative likelihood ratio; PLR: positive likelihood ratio


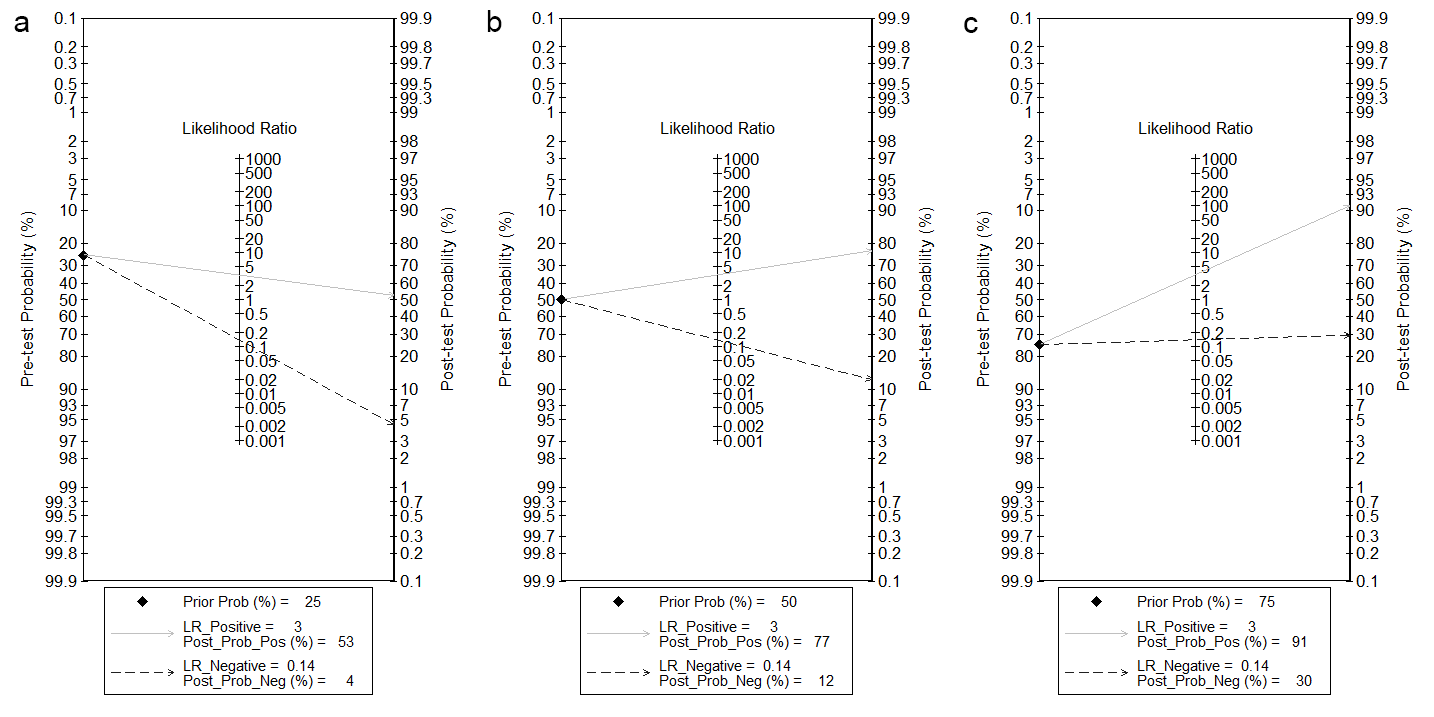


**Supplementary Figure 5 Fagan plot to evaluate the clinical utility of SSM for detecting HREV.**

(a) Pre-test probability=25%; (b) Pre-test probability=50%; (c) Pre-test probability=75%.

NLR: negative likelihood ratio; PLR: positive likelihood ratio


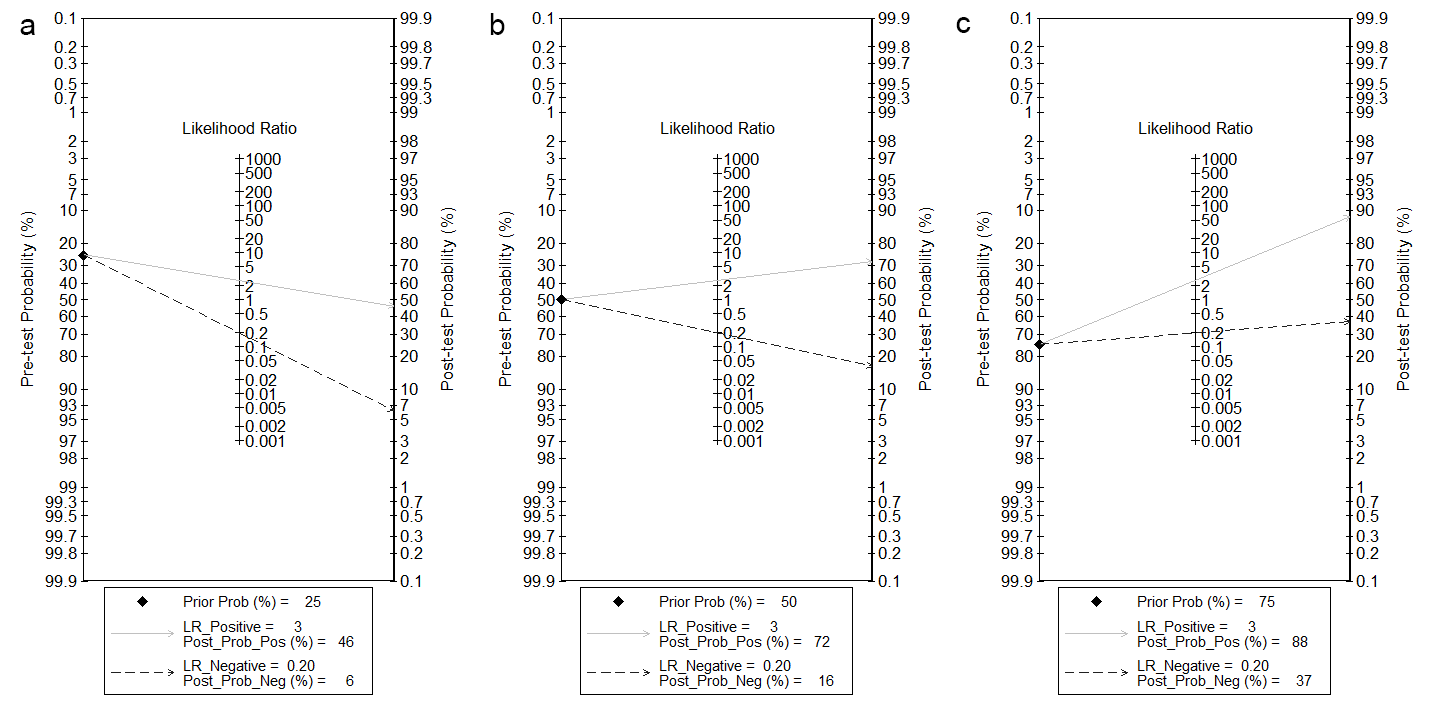

Supplement: Supplementary file 1 — (DOCX 12391 kb) [file 330_2020_7223_MOESM1_ESM.docx]
